# Supplementary material for: Visceral Pleural Invasion as a Determinant of Surgical Strategy in Non–Small Cell Lung Cancer: A Multicenter Study
Source: Cancers (Basel). 2025 Oct 20;17(20):3382. doi: 10.3390/cancers17203382 (PMC12564455; doi:10.3390/cancers17203382)

Supplementary Data

Pathological Visceral Pleural Invasion and Lymph Node Metastasis in Early Stage Non-Small Cell Lung Cancer: A multicenter study

Wakako Nagase[a], Yujin Kudo[a], Takuya Nagashima[b], Takahiro Mimae[c], Yoshihisa Shimada[a], Masaru Hagiwara[a], Masatoshi Kakihana[a] Tatsuo Ohira[a], Yoshihiro Miyata[c], Hiroyuki Ito[b], Morihito Okada[c], Norihiko Ikeda[a]

[a]Department of Surgery, Tokyo Medical University

[b]Department of Thoracic Surgery, Kanagawa Cancer Center

[c]Department of Surgical Oncology, Hiroshima University

Table S1. Patient characteristics of all cases.

Variable	Overall, n = 2,464	Non-adenocarcinoma, n = 350	Adenocarcinoma, n = 2,114	*p-value* (non-Ad vs Ad)
**Age, Median (Minimum, Maximum)**	69 (20,93)	71 (20,93)	68 (23,90)	<0.001[1]
**Sex, n (%)**				<0.001[2]
Men	1,298 (52.7)	284 (81.1)	1,014 (48.0)	
Women	1,166 (47.3)	66 (18.9)	1,100 (52.0)	
**Smoking habit, n (%)**				<0.001[2]
Never smoker	1,090 (44.2)	20 (5.7)	1,070 (50.6)	
Ever smoker	1,368 (55.5)	329 (94.0)	1,039 (49.1)	
Unknown	6 (0.2)	1 (0.3)	5 (0.2)	
**Surgical procedure, n (%)**				0.002[2]
Segmentectomy	663 (26.9)	70 (20.0)	593 (28.1)	
Lobectomy	1,801 (73.1)	280 (80.0)	1,521 (71.9)	
**Tumor location, n (%)**				0.47[2]
Left	979 (39.7)	133 (38.0)	846 (40.0)	
Right	1,485 (60.3)	217 (62.0)	1,268 (60.0)	
**Lymph node dissection, n (%)**				<0.001[2]
Not conducted	74 (3.0)	8 (2.3)	66 (3.1)	
ND1	592 (24.0)	55 (15.7)	537 (25.4)	
ND2a-1	1,542 (62.6)	237 (67.7)	1,305 (61.7)	
ND2a-2	255 (10.3)	50 (14.3)	205 (9.7)	
Unknown	1 (<0.1)	0 (0.0)	1 (<0.1)	

Variable	Overall, n = 2,464	Non-adenocarcinoma, n = 350	Adenocarcinoma, n = 2,114	*p-value* (non-Ad vs Ad)
**Median pathological tumor size, (Minimum, Maximum)**	2.0 (0.2,3.0)	2.0 (0.7,3.0)	2.0 (0.2,3.0)	<0.001[1]
**Visceral pleural invasion, n (%)**				<0.001[2]
Negative	2,094 (85.0)	266 (76.0)	1,828 (86.5)	
Positive	370 (15.0)	84 (24.0)	286 (13.5)	
PL1	254 (10.3)	54 (15.4)	200 (9.5)	
PL2	70 (2.8)	11 (3.1)	59 (2.8)	
PL3	46 (1.9)	19 (5.4)	27 (1.3)	
**Pathological T factor, 9th edition, n (%)**				
Tis	186 (7.5)	0 (0.0)	186 (8.8)	
1mi	389 (15.8)	1 (0.3)	388 (18.4)	
1a	384 (15.6)	16 (4.6)	368 (17.4)	
1b	770 (31.3)	133 (38.0)	637 (30.1)	
1c	341 (13.8)	109 (31.1)	232 (11.0)	
2a	327 (13.3)	69 (19.7)	258 (12.2)	
3	62 (2.5)	18 (5.1)	44 (2.1)	
4	5 (0.2)	4 (1.1)	1 (<0.1)	
**Pathological N factor, 9th edition, n (%)**				<0.001[2]
0	2,165 (87.9)	288 (82.3)	1,877 (88.8)	
N metastasis	296 (12.0)	62 (17.7)	234 (11.1)	
N1	159 (6.5)	42 (12.0)	117 (5.5)	
N2a	104 (4.2)	19 (5.4)	85 (4.0)	

**Variable**	**Overall,**<br>**n = 2,464**	**Non-adenocarcinoma,**<br>**n = 350**	**Adenocarcinoma,**<br>**n = 2,114**	***p-value***<br>**(non-Ad vs Ad)**
N2b	33 (1.3)	1 (0.3)	32 (1.5)	
Unknown	3 (<0.1)	0 (0.0)	3 (<0.1)	
**Pathological stage, 9th edition, n (%)**				
0	186 (7.5)	0 (0.0)	186 (8.8)	
Ⅰ A1	761 (30.9)	17 (4.9)	744 (35.2)	
Ⅰ A2	679 (27.6)	116 (33.1)	563 (26.6)	
Ⅰ A3	266 (10.8)	89 (25.4)	177 (8.4)	
Ⅰ B	229 (9.3)	49 (14.0)	180 (8.5)	
IIA	100 (4.1)	29 (8.3)	71 (3.4)	
ⅡB	151 (6.1)	34 (9.7)	117 (5.5)	
ⅢA	69 (2.8)	13 (3.7)	56 (2.6)	
ⅢB	19 (0.8)	2 (0.6)	17 (0.8)	
ⅣA	1 (<0.1)	1 (0.3)	0 (0.0)	
Unknown	3 (0.1)	0 (0.0)	3 (0.1)	
**Reccurence, n (%)**				<0.001[2]
Non-reccurence	2,190 (88.9)	279 (79.7)	1,911 (90.4)	
Reccurence	274 (11.1)	71 (20.3)	203 (9.6)	

[1]Wilcoxon rank sum test

[2]Pearson's Chi-squared test

[3]Fisher's exact test

Ad, Adenocarcinoma; PL, Pleural invasion

Table S2. Histological types and VPI.

Variable	non-VPI	VPI	% All cases (n = 2464)[1]	% All cases excluding 3 subtypes, (n = 1571)[1]
**Histological subtype of Adenocarcinoma**				
In situ (n = 202, %)	201 (99.5)	1 (0.5)	8.2%	12.9%
Minimally invasive (n = 227, %)	227 (100.0)	0 (0.0)	9.2%	14.4%
Lepidic predominant (n = 464, %)	448 (96.6)	16 (3.4)	18.8%	29.5%
Papillary predominant (n = 708, %)	595 (84.0)	113 (16.0)	28.7%	45.1%
Acinar predominant (n = 261, %)	187 (71.6)	74 (28.4)	10.6%	16.6%
Solid predominant (n = 162, %)	94 (58.0)	68 (42.0)	6.6%	10.3%
Micropapillary predominant (n = 25, %)	20 (80.0)	5 (20.0)	1.0%	1.6%
Mucinous variant (n = 50, %)	45 (90.0)	5 (10.0)	2.0%	3.2%
Others (n = 15, %)	11 (73.3)	4 (26.7)	0.6%	1.0%
**Histological type of non-Adenocarcinoma**				
Squamous cell carcinoma (n = 238, %)	187 (78.6)	51 (21.4)	9.7%	15.1%
Large cell neuroendocrine carcinoma (n = 37, %)	27 (73.0)	10 (27.0)	1.5%	2.4%
Adenosquamous carcinoma (n = 27, %)	16 (59.3)	11 (40.7)	1.1%	1.7%
Pleomorphic carcinoma (n = 19, %)	13 (68.4)	6 (31.6)	0.8%	1.2%
Others (n = 29, %)	23 (79.3)	6 (20.7)	1.2%	1.8%

[1] Percentages are calculated using the same denominator within each column.

VPI, Visceral pleural invasion

Figure S1. Overall survival curves based on VPI and tumor size.

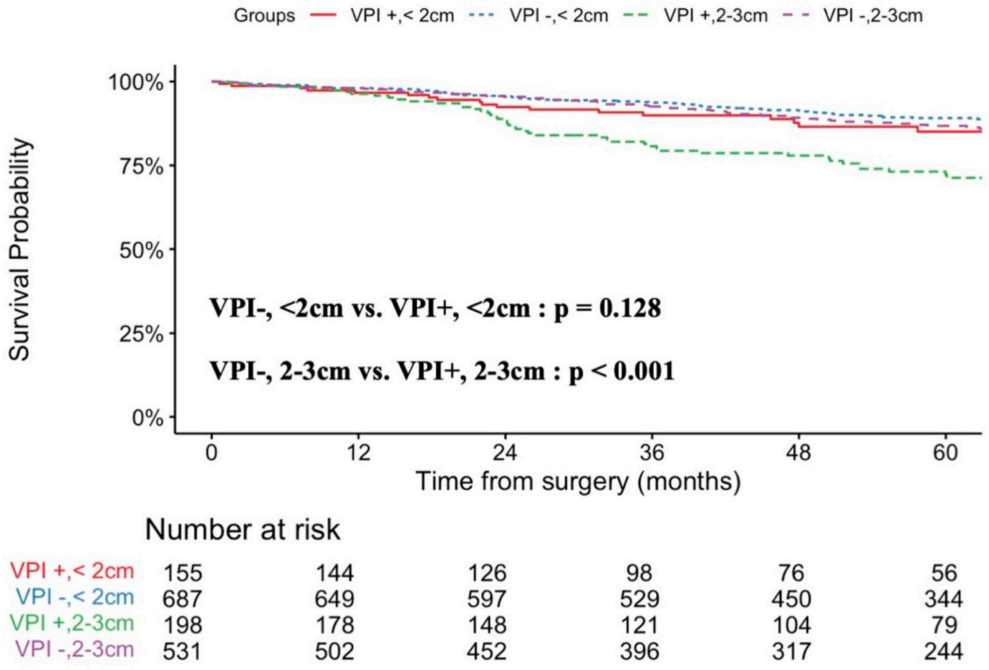

Figure S2. Recurrence-free survival curves based on VPI and tumor size.

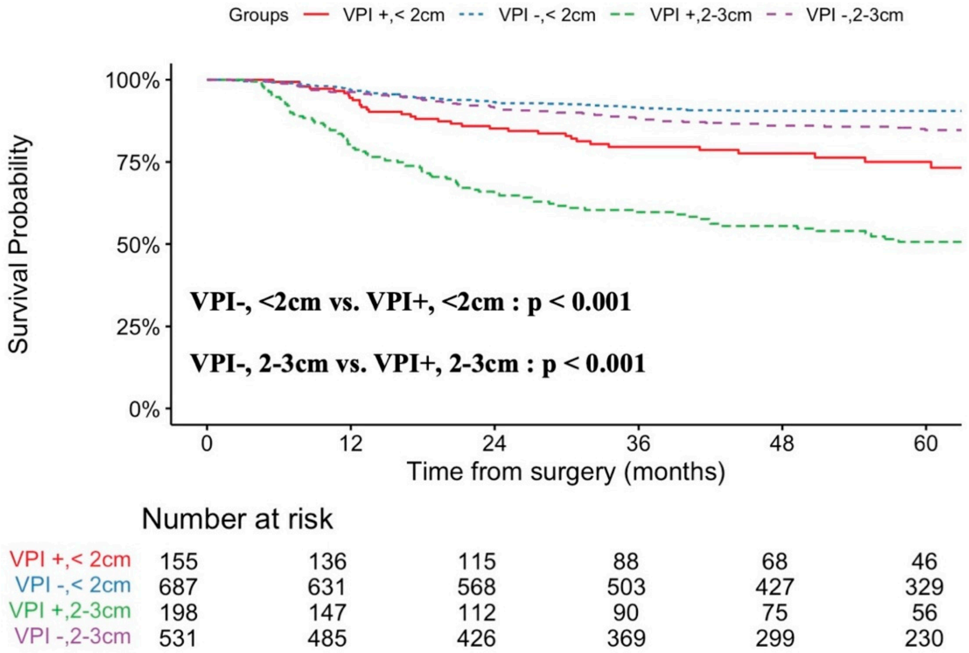

Figure S3. Areas of lymph node metastasis by Histological Type.

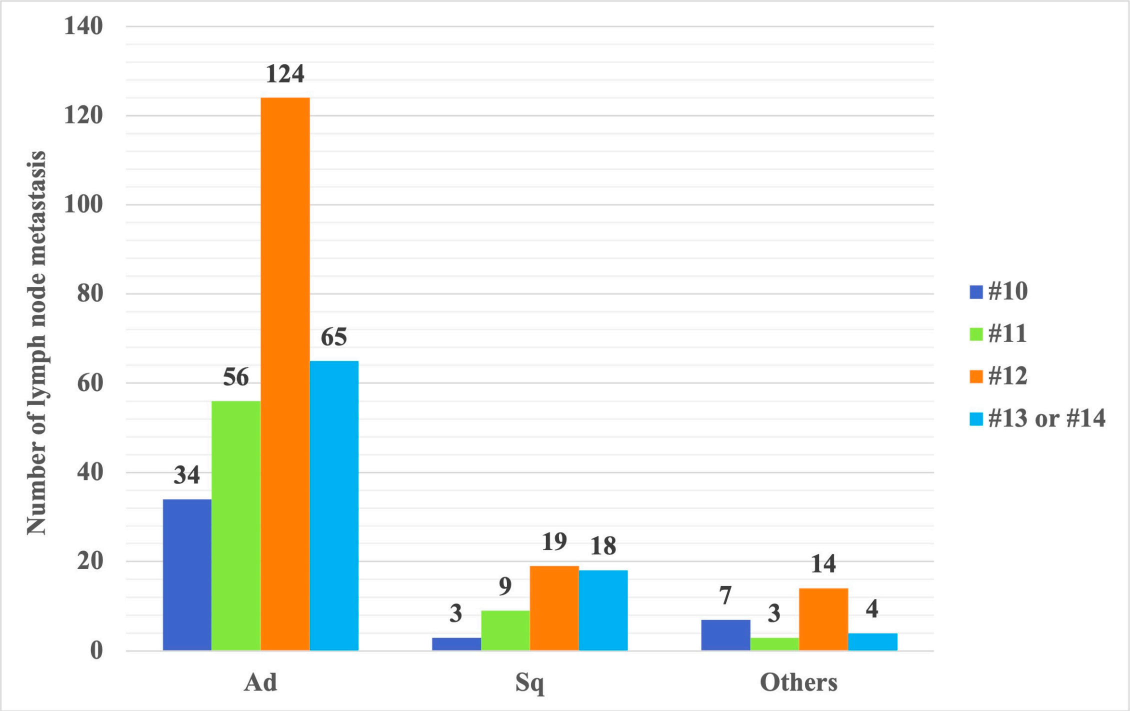

Figure S4. Areas of lymph node metastasis by tumor location.

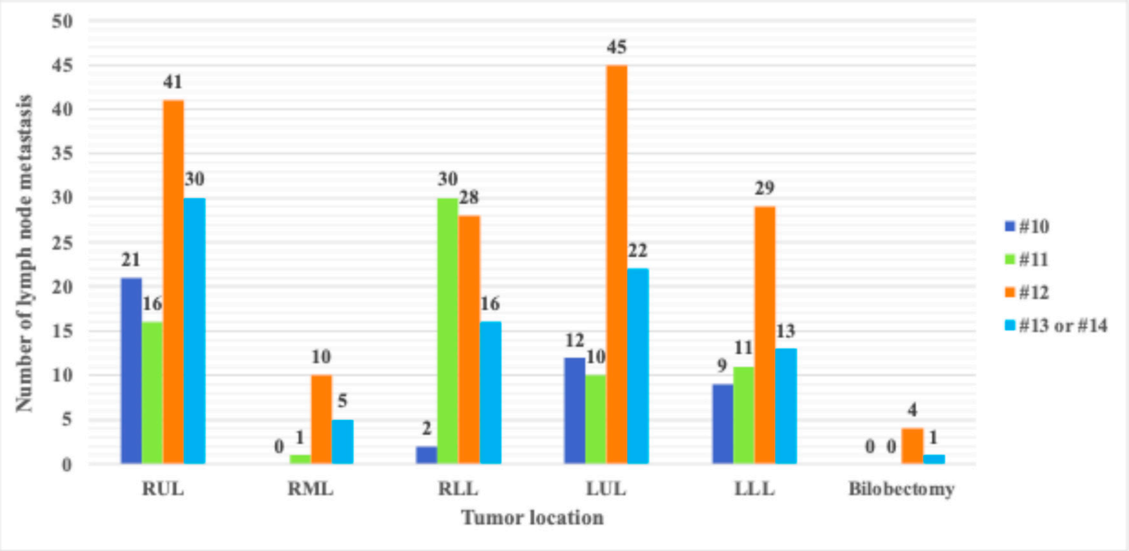

Supplement: Supplementary file 1 [file cancers-17-03382-s001.zip › cancers-3893843-supplementary.pdf]
